# Dental pain and associated factors in Brazilian preschoolers

**DOI:** 10.1016/j.rppede.2016.03.002

**Published:** 2016

**Authors:** João Gabriel Silva Souza, Andrea Maria Eleutério de Barros Lima Martins

**Affiliations:** a Departamento de Ciências Fisiológicas, Faculdade de Odontologia de Piracicaba, Universidade Estadual de Campinas, Piracicaba, SP, Brazil; b Departamento de Odontologia, Universidade Estadual de Montes Claros, Montes Claros, MG, Brazil

**Keywords:** Pain, Child, Tooth decay, Oral health

## Abstract

**Objective:**

To describe the prevalence of dental pain in Brazilian preschoolers, as well as its associated factors, considering a representative sample of that population group in Brazil.

**Methods:**

Cross-sectional study that used the analytical data from national oral health survey (SB Brazil) carried out in 2010. A representative sample of Brazilian preschoolers aged 5 years was considered. Interviews were carried out (answered by parents/tutors), as well as clinical examinations in children. Descriptive, bivariate and mutiple analyzes were performed, described in odds ratios and 95% confidence interval (OR/95%CI).

**Results:**

7280 preschoolers were included. Of these, 1520 (21.1%) had dental pain in the last 6 months. The chance of the occurrence of dental pain was higher among those who used dental services (1.51/1.02–2.23), with tooth decay (3.08/2.08–4.56), that self-perceveid the need for dental treatment (3.96/2.48–6.34), whose parents reported dissatisfaction by children with their teeth and mouth (1.47/1.04–2.10) and those who reported impact of oral problems on quality of life (5.76/3.90–8.49).

**Conclusions:**

The prevalence of dental pain among Brazilian preschool children was relatively high, being associated with the use of dental services and the normative and subjective oral health status.

## Resumo

**Objetivo:**

Descrever a prevalência da dor dentária em pré-escolares brasileiros, assim como seus fatores associados, considerando uma amostra representativa desse contingente populacional no Brasil.

**Métodos:**

Estudo transversal, analítico, que usou os dados do Inquérito Nacional de Saúde Bucal (SB Brasil) feito em 2010. Considerou-se uma amostra representativa de pré-escolares com 5 anos. Foram feitas entrevistas (respondidas pelos responsáveis) e exames clínicos nas crianças. Conduziram-se análises descritivas, bivariadas e múltiplas, descritas em *odds ratio* e intervalo de confiança de 95% (OR/IC95%).

**Resultados:**

Foram incluídos 7.280 pré-escolares. Desses, 1.520 (21,1%) tiveram dor dentária nos últimos 6 meses. A chance da ocorrência de dor dentária foi maior entre aqueles que usaram serviços odontológicos (1,51/1,02-2,23), com cárie dentária (3,08/2,08-4,56), perceberam a necessidade de tratamento odontológico (3,96/2,48-6,34), os que os pais relatavam insatisfação por parte das crianças com seus dentes e sua boca (1,47/1,04-2,10) e os que relataram impacto dos problemas bucais na qualidade de vida (5,76/3,90-8,49).

**Conclusões:**

A prevalência da dor dentária entre pré-escolares brasileiros foi relativamente alta e associada ao uso de serviços odontológicos e às condições normativas e subjetivas de saúde bucal.

## Introduction

Dental pain has been considered the most common symptom or consequence of the presence of oral diseases, such as dental caries and gum disease.^1^^-^ 3 The International Association for the Study of Pain (IASP) defines pain as an unpleasant sensory and emotional experience caused by tissue damage.^4^ Among the types of orofacial pain, those of dental origin have been reported as the most frequent,^5^ may affect social interaction, daily activities,^6^ and may have a negative impact on quality of life.^7^^,^ 8 It should be noted that the perception of pain may be influenced by knowledge and beliefs of the individuals, as well as by the cultural and social environment in which they live.^9^^,^ 10 Different factors have been associated with the presence and perception of dental pain, such as low socioeconomic status,^9^ dental caries,^11^ food-related difficulties, and sleep disorders.^6^ Moreover, its occurrence has been identified as one of the main reasons for seeking dental care.^2^^,^ 9

The prevalence of dental pain varies widely among different studies and age groups. International studies have addressed the prevalence of episodes of dental pain and observed results ranging from 9% in Japan^12^ (11-15 years) to 40% in districts of Manchester, England (up to 12 years).^13^ In Brazil, the prevalence is also variable, with rates between 11% and 39% (subjects aged 5-60 years).^1^^,^ 5 ^,^ 11 ^,^ 14 ^-^ 16 Among the population groups investigated regarding this issue are children, especially those at pre-school age.^8^^,^ 15

In Brazil, the oral health status of preschoolers is worrisome. Despite the modest improvement observed in the last two epidemiological population surveys, conducted in 2002/2003^17^ and 2010,^18^ such as the approximately 6% increase in the number of children under 5 years free of caries, this population group is still affected by a high prevalence of oral diseases, such as dental caries and malocclusion.^18^ This may result in high prevalence of dental pain and, consequently, a negative impact on their daily life.

However, population-based studies with a representative sample of Brazilian preschoolers addressing dental pain are scarce. Therefore, this study aimed to describe the prevalence of dental pain in Brazilian preschoolers and its associated factors.

## Method

This was a cross-sectional study that used the database of the National Survey on Oral Health Conditions (SB Brasil), conducted by the Brazilian Ministry of Health in 2010.^18^ Following the criteria proposed by the World Health Organization in 1997,^19^ a representative sample of the population in the index age ranges was interviewed and examined at their homes regarding their oral health, demographic, and socioeconomic data, as well as use of dental services and subjective issues of oral health. This study considered the sample of preschool children, which in the SB Brazil 2010 included only children aged 5 years.

Residents of 177 cities were interviewed and assessed, including the 27 capitals of the five geographical regions (North, Northeast, Mid-West, Southeast, and South). Subjects were selected by multi-stage probability cluster sampling, with probability proportional to size and considering a design effect (deff) equal to 2. The 30 municipalities in each region and the 30 census sectors for capitals and the Federal District were drawn by the technique of probability proportional to size.^20^

The tests and interviews were performed by previously trained dentists and calibrated by the consensus technique; the minimum acceptable kappa value for each examiner, age group, and studied injury was 0.65. Interviews were conducted with the aid of a handheld computer (Personal Digital Assistant).^20^

In the present study, a database slice was used; the analysis included the preschoolers who answered the question about the presence of dental pain.

The dependent variable - dental pain - was assessed by the question: "in the last six months, did you have toothache?" (No/Yes). Since this was a sample of 5-year-old children, this answer was given by their parents/guardians. Therefore, the presence of dental pain in preschoolers was characterized by the answer "yes".

The independent variables were combined into three groups: sociodemographic conditions, healthcare services, and health outcomes (normative and subjective conditions of oral health). The assessed sociodemographic conditions were sex, self-reported ethnicity, family income, and Brazilian region. Regarding healthcare services, the use of dental care services throughout life was considered. It should be noted that such use, which was defined as having used the service at least once during the lifetime, is not necessarily related to health outcomes or the occurrence of dental pain. Regarding health outcomes, the normative oral health condition assessed was the presence of caries. This assessment was based on the decayed component of the DMFT index, which counts the number of decayed, missing, and filled teeth.^18^^,^ 19 Regarding the subjective conditions of oral health, the self-perceived need for treatment, satisfaction with teeth and mouth, and the impact of oral health on quality of life were assessed. Due to the age of patients, these data were answered by their parents/guardian. This impact was measured by the instrument Oral Impacts on Daily Performance (OIDP), which assesses the impact of oral conditions on the individual's ability to perform their daily activities.^21^^,^ 22 The present study considered as impacted those preschoolers for whom involvement was reported in at least one of the nine items that compose the instrument. Therefore, the dichotomized OIDP score (Yes/No) was used.

For data analysis, SPSS Statistics 18.0 software (SPSS, IBM Company, Hong Kong, China) was used. As the study involved complex cluster sampling, the correction was made by the sample design effect, taking into account the cluster effects and assigning weights to the sampled elements. For categorical variables, the descriptive analysis included the distribution of the sample, corrected relative frequency (%), and standard error (SE). To evaluate the factors associated with the outcome (dental pain), bivariate and mutiple analyses were performed. In the bivariate analysis, the raw odds ratios (OR_raw_) and 95% confidence intervals (95%CI) were estimated, with the correction for the effect of the sample design. Independent variables that presented a descriptive level lower than or equal to 20% (*p*≤0.20) at this stage of the analysis were selected for multiple analysis. In the multiple analysis of the factors associated with the outcome, based on logistic regression (OR_adjusted_/95%CI), a significance level of 5% (*α*=5%) was adopted.

This epidemiological survey was conducted based on the ethical principles of Resolution of the National Health Council No⋅ 196/96 and was approved by and registered at the National Institutional Review Board (Comissão Nacional de Ética em Pesquisa [CONEP]) under No. 15498/2010.

## Results

This study included 7280 children aged 5 years. Of these, 1520 (21.1%) had episodes of dental pain in the six months prior to data collection. Most children were male, had already used dental services, and were caries-free (Table 1).

**Table 1:** Descriptive analysis of dental pain, sociodemographic characteristics, healthcare services, and health outcomes among Brazilian preschoolers in 2010 (*n*=7280).

	*n*	%	SE
*Dental pain in the last 6 months*			
No	5760	78.9	
Yes	1520	21.1	1.7

*Sex*			
Male	3643	51.9	
Female	3637	48.1	1.4

*Ethnicity*			
White	3259	48.5	
Asian/Black/Mixed-race/Indigenous	4021	51.5	2.1

*Family income*			
Over R$ 500	5434	78.1	
Up to R$ 500	1526	21.9	1.8

*Brazilian region*			
Mid-West	1141	8.1	1.1
South	917	13.3	2.1
Southeast	1283	50.3	4.3
Northeast	2145	17.2	2.0
North	1794	11.1	1.4

*Use of dental services*			
No	3416	46.7	
Yes	3800	53.3	1.6

*Tooth decay*			
0	3624	52.6	
1 or more	3535	47.4	1.8

*Self-perceived need for treatment*			
No	3027	45.8	
Yes	3962	54.2	2.0

*Satisfaction with the teeth and mouth*			
Satisfied	4788	71.5	
Unsatisfied	2066	28.5	1.9

*Impact of oral problems in QOL*			
No	5510	74.8	
Yes	1770	25.2	2.2

*n*, number of subjects; SE, standard error; QOL, quality of life.

The bivariate analysis indicated that variables in all categories (sociodemographic, use of services, and health outcomes) remained associated with dental pain (*p*≤0.20) and were considered in the multiple analysis (Table 2).

**Table 2:** Bivariate analysis of factors associated with dental pain in Brazilian preschoolers in 2010.

	Presence of dental pain			
	%	OR_raw_	95%CI	*p*-value
*Sex*				
Male	21.6	1.00		
Female	20.7	0.94	0.72-1.23	0.694

*Ethnicity*				
White	18.5	1.00		
Asian/Black/Mixed-race/Indigenous	23.6	1.36	1.06-1.75	0.16

*Family income*				
Over R$ 500	18.6	1.00		
Up to R$ 500	30.0	1.87	1.39-2.51	0.000

*Brazilian region*				
Mid-West	23.4	1.00		
South	17.4	0.68	0.44-1.06	0.94
Southeast	19.6	0.80	0.55-1.15	0.237
Northeast	24.4	1.5	0.80-1.37	0.688

*Use of dental services*				
No	17.6	1.00		
Yes	24.4	1.51	1.16-1.96	0.2

*Tooth decay*				
0	7.8	1.00		
1 or more	35.5	6.48	4.76-8.83	0.000

*Self-perceived need for treatment*				
No	5.2	1.00		
Yes	35.3	10.2	6.36-15.76	0.000

*Satisfaction with the teeth and mouth*				
Satisfied	12.9	1.00		
Unsatisfied	42.0	4.90	3.76-6.40	0.000

*Impact of oral problems on QoL*				
No	10	1.00		
Yes	54.2	10.63	7.58-14.91	0.000

OR_raw_, raw odds ratio; 95%CI, 95% confidence interval; QOL, quality of life.

In the multiple analysis, dental pain in the last six months in children at 5 years was associated with no use of dental services (*p*=0.037); tooth decay (*p*≤0.001); perception by parents/guardians of their children's need for treatment (*p*≤0.001); parents'/guardians' report of their children's dissatisfaction with teeth and mouth (*p*=0.029); and impact of oral health on quality of life according to the applied instrument (OIDIP - *p*≤0.001; Table 3).

**Table 3:** Multiple analysis of factors associated with dental pain among Brazilian preschoolers in 2010.

	OR_adjusted_	95%CI	*p*-value
*Use of dental services*			
No	1.00		
Yes	1.51	1.02-2.23	0.037

*Tooth decay*			
0	1.00		
1 or more	3.08	2.08-4.56	<0.001

*Self-perceived need for treatment*			
No	1.00		
Yes	3.96	2.48-6.34	<0.001

*Satisfaction with the teeth and mouth*			
Satisfied	1.00		
Unsatisfied	1.47	1.04-2.10	0.029

*Impact of oral problems on quality of life*			
No	1.00		
Yes	5.76	3.90-8.49	<0.001

OR_adjusted_, adjusted odds ratio; 95%CI, 95% confidence interval.

**Figure 1:**
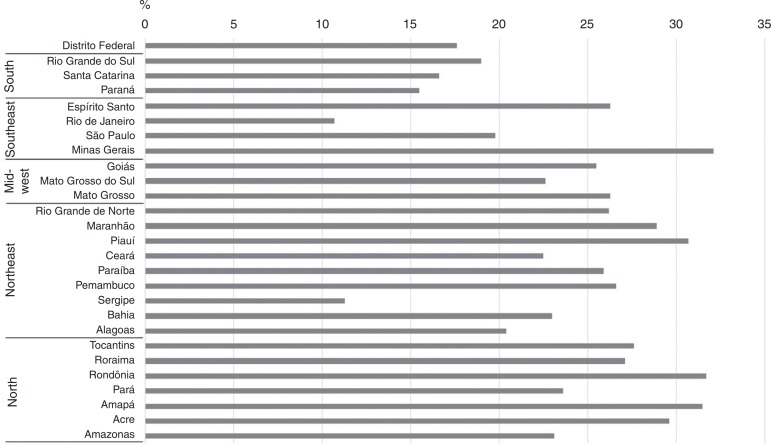
Distribution of the presence (%) of dental pain in Brazilian preschoolers per state.

Regarding the distribution of dental pain among preschoolers, according to the Brazilian states, a lower prevalence of this finding was observed in those living in the South region (Fig. 1).

## Discussion

A high prevalence of dental pain (21.1%) among children aged 5 years was observed. This prevalence was higher than that identified in other studies in Brazil with preschoolers.^15^^,^ 23 Nevertheless, higher prevalences were observed in national^1^^,^ 16 and international^12^^,^ 13 studies that included older children (6-12 years), indicating a possibility that advancing age can influence the occurrence of oral health problems and, consequently, pain perception. The prevalence of dental pain among Brazilian preschoolers possibly derives from the high prevalence of some oral problems among these individuals, such as tooth decay.^18^ However, considering the prevalence of these problems, a higher prevalence of dental pain was expected, since tooth decay has been identified as the primary cause of dental pain in children.^24^ It is worth noting that the report of pain was made by the preschoolers' parents/guardians, who may have underestimated the occurrence of this event. In addition to its rates, it was observed that dental pain was associated with variables related to the use of dental services and outcomes in oral health (normative and subjective conditions of oral health).

This study observed a higher prevalence of dental pain among those who had been to a dentist. This association is worrisome, considering that a dental consultation should stimulate greater care and treatment of dental issues, resulting in pain relief. Thus, it is possible that the dental services used did not resolve the issues. A previous study, conducted among preschool children in Montes Claros (MG - Brazil), identified a lower chance of using dental services among those who had never had dental caries experience,^25^ which evidences the possibility that injuries or pain did not occur, leading to non-use of the service.

Dental caries have been identified as the main cause of tooth pain.^24^^,^ 26 The prevalence of dental pain was higher among preschoolers with one or more teeth with caries. This association was to be expected, since pain is one of the symptoms of tooth decay, and was also identified among adolescents^5^ and adults.^9^ A previous cohort study, with preschool children aged 5 years in Pelotas (RS - Brazil), observed that individuals with caries had a greater chance (4.8 times) of having dental pain.^26^ Increased access to dental services, as well as preventive and health education measures, could have a positive impact on reducing caries rates in this population and, consequently, dental pain.

A greater chance of dental pain was observed in preschoolers who were likely dissatisfied with their oral health conditions and required dental treatment, according to the report by their parents/guardians. The presence of dental pain is a result of the presence of dental injuries^1^^-^ 3 ; these, in turn, can lead to dissatisfaction with oral health and to a perceived need for treatment. It should be noted that the data on subjective issues of oral health were reported by those responsible for the children. Thus, the assessed perception may not fully represent how preschoolers felt affected by their oral problems. However, considering the low age of the sample and the difficulty of assessing their oral conditions, the account of those who live with these children (parents/guardians) is a reliable measure of evaluation. In this sense, dental pain is an important predictor for the search for dental care.

Considering the fact that dental pain negatively impacts people's daily lives^6^ and impairs quality of life,^7^^,^ 8 the greater chance of dental pain among those whose oral health impacted their quality of life identified in this study was expected. The subjective nature of pain reporting, as well as of the impacts on quality of life, especially among children, is noteworthy. Regarding the self-perception of oral health and the need for dental treatment, the assessment of the impact on quality of life was answered by the parents/guardians, and may differ from how the child really felt about their dental problems.

Pain is a multidimensional phenomenon: it can be influenced by different factors, and its objective evaluation in preschoolers is a challenge for healthcare professionals. Therefore, the identification of the prevalence of dental pain and its associated factors may allow for improvements and for the implementation of public policies aimed at fostering better oral health and daily life conditions for this population group. Among the limitations of this study are its transverse design, which does not allow for the identification of causes and effects, and the fact that the data was collected in 2010 and that changes in the pain profile may have occurred over the years. Moreover, the report of pain, as well as of other subjective matters of oral health, was provided by the parents/guardians, and is a subjective and dynamic measurement. Nevertheless, this study allowed for the characterization of the occurrence of dental pain among Brazilian preschoolers, considering a representative sample of this age group. This characterization also allowed for the identification of the distribution of the occurrence of at least one episode of dental pain in preschoolers among the Brazilian states and their capitals. The variability in the prevalence of pain between capital cities of the same region was noteworthy; in general, a lower prevalence was observed in the South of the country.

Of the preschoolers included in this study, 21.1% had dental pain in the last six months prior to data collection. It is noteworthy that this phenomenon remained associated with the use of dental services, dental caries, need for treatment, dissatisfaction with teeth and mouth, and impact of oral health on quality of life. Therefore, such associations should be considered in health planning by healthcare professionals and managers in order to reduce the occurrence of dental pain among Brazilian preschoolers.
